# The Earth’s Heat

**Published:** 1891-12

**Authors:** 


### THE EARTH'S HEAT.

Many scientific men are devoting their lives to finding out all that can be learned about the interior of this wonderful globe of ours. One of the interesting problems on which they are engaged is the depth and geographical limits of permanently frozen soil. The British Association has collected a large amount of data on this question. They have already told us some curious things, such as the fact that excellent wheat lands north of Manitoba overlie frozen earth that never thaws.

Sometimes geologists find strata of rock that they are able to show must have been buried at a remote age, 20,000 feet under the surface. These upturned edges of rock, which some terrible convulsion lifted to the air, give us a glimpse of the condition of the interior some way below the greatest depth to which we can attain. The workmen in the deepest mines of Europe swelter in almost intolerable heat, and yet they have never penetrated over one-seven-thousandth part of the distance from the surface to the center of the earth. In the lower levels of some of the Comstock mines the men fought scalding water, and could labor only three or four hours at a time until the Sutro Tunnel pierced the mines and drew off some of the terrible heat, which had stood at 120 degrees.

The deepest boring ever made, that at Sperenberg, near Berlin, penetrates only 2272 feet, about 1,000 feet deeper than the famous artesian well at St. Louis. The result of this imperfect knowledge is that there are more theories and disputes among scientific men with regard to the interior of the earth than about any other problems of physical science. Some eminent physicists, for instance, like Sir William Thomson, have believed that the crust of the earth is at least 800 miles thick. The majority adduce good reasons for believing that the crust is only twenty-five to fifty miles thick. All agree that if the temperature within the earth continues to increase as it does near the surface—at the rate of one degree Fahrenheit for about every fifty-five feet of descent—all igneous rocks must be fused at no great depth.

In fact, at this rate of increase, the temperature at 200 miles is 18,000 degrees Fahrenheit, which is Professor Rosetti's estimate of the probable temperature of the sun. It is improbable, however, that this rate of increase is maintained for a great distance, and many physicists believe that at some unknown, but not very great depth, the increase in temperature ceases. One of the most wonderful things in the study of sciences is the fact that the mysteries of one science are sometimes completely or partly explained by knowledge gleaned in some other department of study. It is thus that naturalists who have investigated the fauna and flora of scores of Pacific islands have learned how far south Asiatic types prevail, and have added great weight to the conclusions of geologists that these islands were once a part of the big continent north of them.—*Ex.*

